# Assessing Nutritional Deficiencies in Bariatric Surgery Patients: A Comparative Study of Roux-en-Y Gastric Bypass versus Sleeve Gastrectomy

**DOI:** 10.3390/jpm14060650

**Published:** 2024-06-18

**Authors:** José P. Vieira de Sousa, Hugo Santos-Sousa, Sofia Vieira, Rita Nunes, Jorge Nogueiro, André Pereira, Fernando Resende, André Costa-Pinho, John Preto, Bernardo Sousa-Pinto, Silvestre Carneiro, Eduardo Lima-da-Costa

**Affiliations:** 1 Faculty of Medicine, University of Porto, 4200-319 Porto, Portugal; vieira.sousa10@gmail.com (J.P.V.d.S.); sofiavieira97@gmail.com (S.V.); rcsgnunes@hotmail.com (R.N.); nogueiro.jorge@gmail.com (J.N.); andre.d.a.pereira@gmail.com (A.P.); fernandosilvaresende@gmail.com (F.R.); andrecostapinho@gmail.com (A.C.-P.); bernardo@med.up.pt (B.S.-P.); silvestre.carneiro@gmail.com (S.C.); elimadacosta@gmail.com (E.L.-d.-C.); 2 Surgery Department, São João University Medical Center, 4200-319 Porto, Portugal; 3 Obesity Integrated Responsibility Unit (CRI-O), São João University Medical Center, 4200-319 Porto, Portugal; jrpreto@gmail.com; 4 MEDCIDS—Department of Community Medicine, Information and Health Decision Sciences, Faculty of Medicine, University of Porto, 4200-450 Porto, Portugal; 5 CINTESIS—Center for Health Technology and Services Research, 4200-450 Porto, Portugal

**Keywords:** obesity, nutritional deficiencies, bariatric surgery, Roux-en-Y gastric bypass, sleeve gastrectomy

## Abstract

Obesity is a worldwide epidemic, and bariatric surgery is considered the primary treatment for long-term weight loss and managing obesity-related health issues. Sleeve gastrectomy (SG) and Roux-en-Y gastric bypass (RYGB) are the most performed procedures. Nutritional deficiencies are a significant concern following bariatric surgery and can have serious consequences. This study aims to compare the incidence of nutritional deficiencies in patients undergoing RYGB and SG. A retrospective analysis was conducted on the nutritional status of 505 consecutive patients who underwent either RYGB or SG between January and December 2019. Data were collected regarding vitamin B12, folic acid, vitamin D, calcium, PTH, magnesium, hemoglobin, iron, ferritin, and transferrin at preoperative, 6-month, and 12-month intervals post-surgery. The RYGB group showed significantly higher excess weight loss. Vitamin B12, hemoglobin, and ferritin levels were consistently higher in the SG group throughout the study. Vitamin D deficiency was prevalent, with no significant difference between the groups. Vitamin B12 deficiency was significantly more common in the RYGB group (6 months: 17.46% vs. 4.69%, *p* < 0.001; 12 months: 16.74% vs. 0.93%, *p* < 0.001). Despite differences in their mechanisms, bariatric surgeries were associated with nutritional deficiencies. It is crucial to efficiently assess, prevent, and manage these deficiencies tailored to each surgical procedure.

## 1. Introduction

Obesity has emerged as a significant global healthcare challenge in recent decades, now recognized as an epidemic of the twenty-first century [1]. By 2022, 43% of the adults worldwide were classified as overweight and 16% as obese, with a higher prevalence among women than men [2]. In Europe, the prevalence of obesity among adults ranges from 10% to 30% [3].

The increase in obesity can be linked to various factors contributing to an “obesogenic” environment: industrialization and fast economic growth, the increasingly sedentary lifestyle, and the consumption of low-cost processed foods high in calories but low in nutrients [4,5].

Overweight and obesity are major risk factors for numerous diseases, such as cardiovascular conditions [6], diabetes [7], and even some cancers [8], leading to substantial morbidity and mortality. Consequently, effective treatment is crucial, with bariatric surgery recognized as the most effective intervention [9,10,11]. Sleeve gastrectomy (SG) and Roux-en-Y gastric bypass (RYGB) are the two most performed techniques [12,13,14], both proving similarly effective in reducing weight [13,15].

As the obese population continues to grow, the number of bariatric surgeries performed worldwide has also increased. In 2019, an estimated 256,000 bariatric surgeries were performed in the United States of America [12], while in Europe, data from The International Federation for the Surgery of Obesity and Metabolic Disorders (2019) reported 381,627 surgeries from 2014 to 2019 [14].

Despite being an essential tool in combating obesity, bariatric surgery is not without its complications. One of the most significant side effects of this technique is the development of nutritional deficiencies or the exacerbation of preexisting ones. This is particularly critical as obesity itself is associated with a high prevalence of nutritional deficiencies, potentially leading to devastating consequences for patients [16].

The underlying variables associated with postoperative vitamin and mineral deficiencies are surgery- and patient-related. Preoperative nutritional deficits, altered eating habits, decreased absorption surface, small intestine bacterial overgrowth, and poor compliance with the postoperative recommendations for diet optimization and nutritional supplementation are associated with the nutrient-deficient state [17]. Food intake restriction, decreased appetite, and gastrointestinal hormone profile changes are common mechanisms for weight loss reported after RYGB and SG, affecting nutrient status as well. The malabsorptive aspect of RYGB impairs vitamin and mineral absorption because the remnant stomach and the upper part of the small intestine are excluded from the gastrointestinal transit [18]. The degree of malabsorption is related to the length of the common channel rather than the lengths of the Roux limb. Since the small bowel remains intact after surgery, one would expect the report of fewer micronutrient deficiencies after SG than RYGB. However, several recent reports show that micronutrient deficiencies may occur to the same extent after both types of surgery despite intact intestinal absorptive surface area in SG. SG impacts micronutrient status by altering gastrointestinal motility, accelerating gastric emptying and gastro-duodenal transit time, and decreasing hydrochloric acid and intrinsic factor secretion [17].

Although there are already some studies in this area, the results remain heterogeneous due to variations in assessed nutrients, cut-off values, follow-up periods, and supplementation strategies across institutions. The most common micronutrient deficiencies after RYGB and SG are vitamin B12, folic acid, iron, thiamine (vitamin B1), vitamin D, and calcium. Other reports on nutritional deficiencies after weight loss surgery mention fat-soluble vitamins (vitamins A, E, and K), as well as copper, zinc, and selenium [17].

Several studies are aligned with this finding, reporting that de novo deficiency is uncommon, and only identifying a higher risk for deficiency in some key nutrients. Although prophylactic micronutrient supplementation is still required, these findings question the need for a high dose of all ten nutrients contained in the prophylactic multivitamin prescriptions after SG or RYGB, especially where the limb length is ≤150 cm and patients are supported in a multidisciplinary postoperative setting [19]. International guidelines recommend lifelong vitamin and mineral supplementation and nutritional biochemical monitoring post-surgery, tailored to the specific bariatric procedure. For instance, guidelines from the British Obesity and Metabolic Surgery Society (BOMSS) and the Enhanced Recovery After Surgery (ERAS) Society emphasize comprehensive supplementation and frequent monitoring to prevent deficiencies [20,21]. These guidelines highlight the importance of individualizing supplementation protocols based on patient needs and surgical variables.

Therefore, our study aimed to compare the incidence of nutritional deficiencies in obese patients undergoing SG and RYGB at our institution to improve patient management and mitigate the risk of deficiencies in the future.

## 2. Materials and Methods

### 2.1. Study Design and Participants

This study is a retrospective analysis of 505 consecutive patients who underwent bariatric surgery at our institution between 1 January 2019 and 31 December 2019. Our hospital serves as a referral center for bariatric patients and performed 580 surgeries in 2019. We excluded the patients who underwent gastric band removal surgeries, complications-related surgeries (*n* = 60), as well as those who underwent single anastomosis duodeno-ileal bypass with sleeve gastrectomy, one anastomosis gastric bypass, silastic ring gastric bypass, and the revision of RYGB with limb distalization (*n* = 15).

Thus, we reviewed the electronic medical records of 165 patients submitted to SG and 340 patients who underwent RYGB. Table 1 provides a comparison of the characteristics of the RYGB and SG groups.

The eligibility criteria for bariatric surgery include patients between 18 and 65 years old with BMI ≥ 40 kg/m^2^ or BMI ≥ 35 kg/m^2^ with obesity-related comorbidities [13,24]. The exclusion criteria for surgery include the absence of one year of conservative treatment, the inability to follow-up, psychiatric disorders, drug or alcohol abuse, short-term fatal diseases, and patients incapable of self-care or lacking social support.

### 2.2. Surgical Techniques

This study compared RYGB and SG, with both the procedures standardized and uniformly performed throughout the study period.

RYGB involved creating a calibrated gastric pouch (using a 36-Fr Fouchet’s bougie), positioning the roux limb 100 cm distally to the ligament of Treitz, and performing the jejunojejunostomy 120 cm distally to the gastrojejunostomy.

In the SG technique, stomach resection was performed with an Echelon^TM^ 60 mm, with calibration achieved using a 54-Fr Fouchet’s bougie.

### 2.3. Follow-Up

The patients in our institution undergo follow-up every six months during the initial two years after surgery, followed by annual visits thereafter. Each follow-up visit evaluates weight loss, comorbidities, and nutritional status.

This study analyzed the values of vitamin B12, folic acid, vitamin D, calcium, parathormone (PTH), magnesium, hemoglobin, iron, ferritin, and transferrin at three different intervals: preoperatively, and 6 and 12 months after surgery. Deficiency thresholds (or excess in the case of PTH and transferrin) were determined according to the hospital laboratory reference ranges, listed in Table 2. Vitamin D deficiency is defined as a 25(OH)D level of less than 20 ng/mL, while vitamin D insufficiency is recognized as a 25(OH)D level of 21–29 ng/mL. The recommended goal is to maintain 25(OH)D levels above 30 ng/mL to take full advantage of all the health benefits that vitamin D provides [25].

After the surgery, the patients were advised to take daily multivitamin supplementation (1 to 2 tablets of Centrum^®^: 800 µg of vitamin A, 15 mg of vitamin E, 100 mg of vitamin C, 30 µg of vitamin K, 1.4 mg of thiamine, 1.75 mg of riboflavin, 2 mg of vitamin B6, 2.5 µg of vitamin B12, 5 µg of vitamin D, 62.5 µg of biotin, 200 µg of folic acid, 162 mg of calcium, 125 mg of phosphorus, 100 mg of magnesium, 5 mg of iron, 100 µg of iodine, 500 µg of copper, 2 mg of manganese, 40 µg of chromium, 50 µg of molybdenum, 30 µg of selenium, and 5 mg of zinc).

### 2.4. Statistical Analysis

Statistical analysis was performed using SPSS^®^ version 26.0 for Mac (IBM Co., Armonk, NY, USA). Continuous variables were presented as median values with interquartile range, while categorical variables were expressed as percentages.

To compare the two techniques, we applied the chi-squared test and the independent samples Mann–Whitney U test. The statistical significance level was set at *p*-values < 0.05.

## 3. Results

Table 1 displays the anthropometric characteristics of the two groups, totaling 505 patients, who were followed for 12 months. Most participants in both the groups were women, accounting for 88.82% and 72.73% of the RYGB and the SG samples, respectively. At the baseline, the median age of the patients was 46 years in the RYGB group and 45 years in the SG group.

The BMI was significantly higher in the SG group both preoperatively and throughout the entire 12-month follow-up period compared to the RYGB group. Similarly, the RYGB group exhibited significantly higher excess weight loss at 6 and 12 months after surgery.

There were no significant differences in comorbidities between the groups, with diabetes mellitus being more prevalent in the RYGB group, while hypertension and dyslipidemia predominated in the SG group.

When comparing the median value of the measured parameters (Table 3), vitamin B12, hemoglobin, and ferritin were the only ones that showed significant differences between the two groups throughout the study period. Additionally, differences in the nutrient levels were observed at various points during the study: folic acid and transferrin were significantly higher in the RYGB group at 12 months of follow-up, calcium was higher in the SG patients throughout the postoperative period, and PTH and iron levels were higher in the RYGB and the SG groups, respectively, at 6 months after surgery.

The deficiencies of each nutrient are outlined in Table 4. Notably, vitamin D deficiencies were prevalent preoperatively in both groups, with no significant difference between them: 62.05% in the RYGB and 74.24% in the SG group. In the postoperative period, these deficiencies became more prevalent in the RYGB patients at 6 months after surgery (29.64% vs. 24.03%), and in the SG group at 12 months (31.48% vs. 22.41%).

Statistically significant differences between the two types of surgery were observed in vitamin B12 deficiency post-surgery, with a higher prevalence in the RYGB patients (17.56% vs. 4.96% at 6 months and 16.74% vs. 0.93% at 12 months). Additionally, the RYGB group exhibited higher levels of excess PTH at six months post-surgery (16.40% vs. 8.66%) and excess transferrin at 12 months post-surgery (5.15% vs. 0%) compared to the other group.

Calcium deficiencies were more prevalent in the RYGB group before and 6 months after surgery, shifting to a higher prevalence in the SG group after 12 months (although this difference was not significant).

Magnesium deficiencies were relatively prevalent in both groups, although the difference between the groups was not significant. Magnesium deficiencies were slightly more common in the RYGB group throughout the entire follow-up period. Conversely, folic acid deficiencies were rare.

Anemia, iron, and ferritin deficiencies, as well as transferrin excess, were more prevalent in the RYGB group, except for iron deficiency, which was higher in the SG group preoperatively (15.79% vs. 12.69%).

Moreover, it is noteworthy that before surgery, 73.82% of the RYGB patients and 78.18% of the SG patients were under some supplementation to correct specific deficiencies.

## 4. Discussion

The study results revealed several nutritional deficiencies in bariatric patients, either in the pre- or postoperative periods.

### 4.1. Vitamin B12

The most considerable difference between the patients who underwent RYGB or SG was observed in relation to vitamin B12, particularly after the surgery. The RYGB group demonstrated a 17.46% and 16.74% prevalence of vitamin B12 deficiency at 6 and 12 months, respectively, while the SG group exhibited lower rates of 4.69% and 0.93%, respectively. Consistent with our findings, systematic reviews by Alexandrou et al., Antoniewicz et al., and Kwon et al. showed that SG carries a lower risk of postoperative vitamin B12 deficiency compared to RYGB [26,27,28]. On the other hand, Ferraz et al. did not find any significant difference between the two surgical techniques concerning vitamin B12 deficiency [29]. Preoperative vitamin B12 deficiency in our study population aligned with the reports from the American Society for Metabolic and Bariatric Surgery [16].

Vitamin B12 absorption involves a complex series of metabolic steps within the gastrointestinal tract. Humans require a dietary source of vitamin B12 due to the complexity of its biosynthesis, which is limited to prokaryotes [17]. The intrinsic factor (IF), synthesized by the parietal cells of the stomach, plays a crucial role in cobalamin absorption [30,31]. In RYGB patients, there are several potential mechanisms that may contribute to vitamin B12 malabsorption and deficiency: (1) the decreased acid and pepsin digestion of protein-bound cobalamins from food, (2) the incomplete release of vitamin B12 from R proteins due to inadequate mixing of nutrients with pancreatic secretions, and (3) the decreased availability of IF [17]. Increased intolerance to certain foods, such as meat, was also identified as a potential exacerbating factor for these deficiencies [32]. Furthermore, Majumder et al. also noted that bacterial overgrowth could contribute to the vitamin B12 deficit [33].

Changes in the architecture of the gastrointestinal tract secondary to gastric fundus resection result in decreased hydrochloric acid and pepsin secretion by the functional remnant segment [30,34,35]. This leads to the inadequate capture of vitamin B12 from dietary sources and the lack of food contact with the IF-producing cells, thus causing cobalamin malabsorption and deficiency. The small pouch constructed from the gastric cardia in the RYGB procedure is virtually devoid of acid secretion, causing food-bound vitamin B12 to be maldigested and subsequently malabsorbed [17].

A small study showed a decreased gastric production of transcobalamin 1 (TCN1) after RYGB, affecting B12 intestinal transport. Cobalamin preferentially binds to TCN1 in the low acidic gastric environment and attaches to IF only in the small upper bowel. Despite indirect improvements in cobalamin absorption following bariatric procedures, studies show a higher incidence of vitamin B12 deficiency after RYGB than SG. The authors argue that after restrictive bariatric surgery, TCN1 synthesis might be inhibited; however, food exposure to the entire intestinal surface area leads to local IF production and IF-mediated B12 gut transport is less affected [28].

Recommendations for preventing vitamin B12 deficiency after bariatric surgery include the oral intake of 350–500 mg per day or the intramuscular administration of 1000 mg per month [16,36]. Vitamin B12 is a complex biomolecule that plays a vital role in key biochemical reactions. As a water-soluble vitamin derived from dietary sources such as eggs, red meat, and dairy, it is not stored in large quantities, and excess amounts are rapidly eliminated from the body. Both oral and intramuscular (IM) vitamin B12 are common routes for treating B12 deficiency, with several studies evaluating their efficacy. Deficiency is mainly caused by inadequate dietary intake. Severe clinical abnormalities require intensive treatment with B12, such as cyanocobalamin or hydroxocobalamin. Following an intramuscular injection of 1000 μg of cyanocobalamin, approximately 150 μg is retained in the body, primarily stored in the liver, although retention variability is high across studies. Hydroxocobalamin, commonly used in Europe, is often administered at intervals of 2 to 3 months due to its better retention than cyanocobalamin. High-dose oral supplementation is an effective alternative to parenteral treatment. Studies have shown that 0.5–4% of an oral B12 dose is absorbed; thus, high-dose tablets of 1000 μg provide, on average, 5–40 μg of B12. Randomized studies have demonstrated that the daily oral doses of 1000–2000 μg are equivalent or superior to injected B12 in maintaining adequate levels. In summary, while the parenteral administration of 1000 μg of B12 monthly may not provide a consistent supply due to the rapid excretion of excess amounts, the daily oral administration of 350–500 μg, or even higher doses, is more effective in maintaining stable and adequate vitamin B12 levels. This approach is particularly beneficial for patients with compromised absorption capacities, such as those who have undergone bariatric surgery [37].

In our center, the current supplement prescribed to our patients contains less than the recommended dose for this patient population. Therefore, it is imperative to consider developing specialized supplementation tailored to the specific needs of bariatric patients based on the type of surgery they undergo. This approach aims to mitigate the prevalence of vitamin B12 deficiencies, particularly in the RYGB group.

### 4.2. Folic Acid

Our study revealed that folic acid deficiency was negligible at 6 months post-surgery and remained rare at 12 months, consistent with the findings from other reported studies [35]. This can be attributed to the adaptive mechanisms of folate absorption in the entire small bowel following the bariatric surgery, rather than being absorbed in the proximal segment as usual [26,30].

### 4.3. Vitamin D

This study reveals that vitamin D was the most common nutrient deficiency both before and after surgery, with prevalence rates of 62.05% and 74.24% in the RYGB and SG groups, respectively. This is consistent with previous research on the Mediterranean population [38]. The high deficiency rates may be due to the body storing lipid-soluble vitamins in adipose tissue, reduced sunlight exposure, and the low intake of dairy products [5,35,39]. After surgery, the deficiency rates decreased significantly, with fluctuations between the RYGB and SG groups at 6 and 12 months post-surgery. The altered anatomy in the RYGB procedure can lead to the decreased absorption of fat-soluble proteins because of the delayed blend of the food with bile and pancreatic enzymes [40]. In our study, the high prevalence of hypovitaminosis D before surgery may help explain the lack of significant differences between the groups after surgery.

### 4.4. Calcium and PTH

Calcium deficiency was rare in our study and did not show any significant difference between the RYGB and SG groups. It was more common in the RYGB group before and at 6 months after surgery, but after 12 months it became more frequent in the SG patients. This can be explained by the fact that procedures like RYGB create a bypass to the duodenum and jejunum, which are the preferential sites for calcium absorption. Additionally, insufficient levels of vitamin D can limit the transcellular vitamin D-dependent active transport of calcium. Therefore, the increase in the calcium deficiency at 12 months in the SG group may be related to the simultaneous increase of the vitamin D deficiency in this same group [36,40]. Furthermore, the intolerance of dairy products might contribute to this deficiency [32].

We also assessed the levels of PTH, which is a crucial indicator of calcium metabolism [41]. After undergoing RYGB surgery, the patients showed a significant increase in PTH levels, with 16.40% of these patients having a value above the normal range (vs. 8.66% in the SG group) at 6 months of follow-up. In this same period, calcium and vitamin D deficiencies were also more prevalent in the RYGB group, revealing an intensification in bone metabolism due to the development of secondary hyperparathyroidism [42,43]. As this can lead to bone mineralization issues (such as osteopenia or osteoporosis), it is essential to maintain the long-term screening and supplementation of vitamin D and calcium [43,44,45,46].

### 4.5. Magnesium

Regarding magnesium, the study found that the RYGB patients experienced more deficiencies throughout the study, although without statistical difference. Hypomagnesemia has been linked to insulin resistance and metabolic syndrome, so addressing this deficiency could be an essential step to improve patients’ health [47].

### 4.6. Hemoglobin, Iron, Ferritin, and Transferrin

The prevalence of anemia in our study increased after surgery in both the groups, with a higher prevalence in the RYGB patients, as demonstrated by Antoniewicz et al. [27] and Ferreira et al. [48]. However, there was no significant difference between the two surgical techniques. Although vitamin B12 deficiency can lead to changes in erythropoiesis, iron deficiency remains one of the most frequent causes of anemia [49], as seen in our patients throughout the study period (Table 4). Before surgery, the constant inflammatory state of obesity with the elevated production of hepcidin, along with poor dietary intake, led to a reduction in iron absorption, which may justify the iron deficiency in these patients [26,35,50,51].

The inflammatory state also explains the lower prevalence of ferritin deficiency compared to iron deficiency, as ferritin is a positive acute-phase protein [52]. Also, we found a lower prevalence of iron deficiency in the postoperative period compared to pre-surgery, along with an increase in ferritin deficiencies, likely due to reduced inflammation because of weight loss.

After surgery, the RYGB group showed a higher prevalence of iron and ferritin deficiencies and transferrin excess. This could be attributed to factors such as bypassing the major sites of the absorption of iron (duodenum and proximal jejunum), reduced stomach acid, and decreased meat consumption [26,32,35,52,53]. Additionally, our sample consisted mostly of women of reproductive age, which could exacerbate iron deficiency, especially after surgery, as weight loss can help resolve potential menstrual irregularities in obese women [52].

The study findings indicate that nutrient deficiencies were more prevalent before surgery, except for vitamin B12, hemoglobin, and ferritin. This could be attributed to the patient’s pre-surgery diet, which was high in energy but lacking in various nutrients, as well as the use of restrictive diets that may contribute to rapid metabolic changes and exacerbate these deficiencies [35,54]. After surgery, the patients received more rigorous follow-up care and were supplemented with a multivitamin, leading to a decrease in the prevalence of deficiencies in the postoperative period.

Although there are no strict guidelines for correcting pre-surgery deficiencies, our center emphasized the assessment and correction of nutritional deficiencies before surgery due to evidence suggesting that surgery may exacerbate these deficiencies [26,55,56]. In our sample, 73.82% of the RYGB patients and 78.18% of the SG patients received supplementation before surgery. It is important to note that obesity itself can cause nutritional deficiencies, especially in iron and vitamin D, due to the inflammatory process associated with excess weight. Higher levels of ferritin have been observed in obese subjects than in non-obese individuals. This is probably related to the low-grade chronic inflammation that characterizes obesity. However, despite the higher levels of ferritin, the obese subjects present lower serum iron (Fe) concentrations and reduced percentage of transferrin saturation compared with the non-overweight subjects, and therefore the prevalence of iron deficiency is significantly higher in the obese individuals. The reduction in serum Fe is partially explained by chronic inflammation accompanying obesity, which sets in motion a series of pathological mechanisms. Moreover, the liver is frequently affected by lipid accumulation, called non-alcoholic fatty liver disease (NAFLD), which contributes to the disturbance of iron balance by increasing cytokine production and developing insulin resistance [57]. A meta-analysis concluded that obesity is associated with an increased risk of vitamin D insufficiency or deficiency, independent of latitude, age, and conditions of human development. It has been suggested that excess body fat retains vitamin D metabolites, sequestering cholecalciferol produced through the skin or diet before it reaches the liver for hydroxylation. The elevated levels of the vitamin D activation enzyme 1-α-hydroxylase in the adipose cells of obese individuals may explain the increased local utilization of 25(OH)D. Variations in serum 25(OH)D and vitamin D reserves are related to the amount of subcutaneous fat. Wortsman et al. reported that, following sunlight exposure, the serum concentration of 25(OH)D increased by 53% less in obese individuals than non-obese individuals [58,59].

In our practice, RYGB is the preferred procedure. However, in patients at the extremes of age and BMI, SG is performed. Because our department has a super obesity rate of 10–15%, the SG group will statistically have a higher baseline BMI than the RYGB group, thus possibly introducing a selection bias. In super-obese patients, SG is preferred because of the technical challenges of performing RYGB in these patients. Also, SG is frequently used as a bridge procedure, allowing the patients to reduce weight initially, thus making other procedures safer and more effective later.

Proton pump inhibitors are known to increase the risk for vitamin and mineral deficiencies and to affect vitamin B12, vitamin C, calcium, iron, and magnesium metabolism. Although the risk is low in the general population with the use of these medications, it may be considerable in the elderly and malnourished patients [60]. All the patients received PPIs for the first 2 months following surgery, up to the time of their first follow-up visit. After 6 months, PPIs were discontinued if no symptoms of reflux were present [20].

However, the study had several limitations, such as its retrospective nature, single-center design, challenges in accurately assessing post-surgery supplementation compliance, and the impact of the COVID-19 pandemic, which led to the cancellation of many follow-up visits and laboratory evaluations. Additionally, the study used serum vitamin B12 instead of serum methylmalonic acid to identify deficiencies [16]. Likewise, we did not study the mean corpuscular volume in the patients with anemia to establish potential correlations with vitamin B12 and iron deficiencies. As this study was conducted in a high-volume specialized center with experienced surgeons and more complex cases, its applicability to other contexts may be limited.

The study emphasized the importance of patient adherence to supplementation, with all the patients being encouraged to take multivitamins and their adherence closely monitored. Continuous efforts were made to enhance patient adherence during each follow-up, conducted every six months in the first year and annually after that. Additionally, at each follow-up appointment, supplementation was adjusted based on signs or symptoms of nutritional deficits and analytical changes. However, a limitation of this study is the lack of objective data on adherence levels and on the patients who developed signs or symptoms of nutritional deficit, representing a limitation of our study.

Nonetheless, a strength of our study lies in the analysis of a large sample of 505 patients over three different time points, providing comprehensive insights into changes in nutritional status throughout the follow-up period across ten different analytical parameters. We recognize that the follow-up period in this study was relatively short. However, we chose to conduct this study over this period to emphasize the early and short-term postoperative outcomes of the surgery on nutrition. This approach allows us to closely monitor the initial impact of the surgery and the early occurrence of nutritional deficiencies. Future studies with longer follow-up periods are planned to comprehensively assess the long-term outcomes of the surgical procedures.

The fact that our study was limited to RYGB and SG procedures and that we excluded other less standardized techniques in our center might reduce potential bias.

## 5. Conclusions

Consistent with other findings, our research revealed that the patients with obesity who undergo bariatric surgery develop a range of nutritional deficiencies, reflecting the intricate interplay between nutrient metabolism and specific surgical procedures. Notably, vitamin D deficiency is common in bariatric surgery patients, and vitamin B12 deficiency is higher in the patients undergoing RYGB.

Healthcare professionals working with obese patients should be mindful of these issues, as they can have far-reaching effects. The assessment of nutritional status before surgery and rigorous postoperative monitoring of these patients is mandatory, along with tailored supplementation strategies based on individual needs and the type of surgery performed.

Further research is needed to develop comprehensive guidelines that address these complexities.

## Figures and Tables

**Table 1:** Comparison of demographic characteristics, clinical parameters, and comorbidities between Roux-en-Y gastric bypass and sleeve gastrectomy patients.

Characteristics	Roux-en-Y Gastric Bypass *n* = 340	Sleeve Gastrectomy *n* = 165	*p*-Value
Gender, *n* (%)			<0.001
Female	302 (88.82%)	120 (72.73%)	
Male	38 (11.18%)	45 (27.27%)	
Age at surgery (years), median (IQR ^1^)	46.00 (40.00–54.00)	45.00 (36.00–54.50)	0.400
Body mass index (kg/m^2^), median (IQR ^1^)			
Before surgery	42.31 (39.25–45.34)	43.82 (39.80–49.12)	0.005
6 months post-surgery	30.55 (27.85–33.15)	32.21 (28.72–35.31)	<0.001
12 months post-surgery	27.41 (25.23–29.76)	29.78 (26.22–33.67)	<0.001
Percentage of excess weight loss ^1^, median (IQR ^2^)			
6 months post-surgery	67.66 (56.82–82.32)	61.77 (49.27–74.16)	0.001
12 months post-surgery	86.07 (75.12–98.34)	74.82 (62.80–92.90)	<0.001
Comorbidities, *n* (%)			
Diabetes mellitus	95 (27.94%)	36 (21.82%)	0.141
Arterial hypertension	158 (46.47%)	82 (49.70%)	0.496
Dyslipidemia	143 (42.06%)	70 (42.42%)	0.938

^1^ the percentage of excess weight loss (%EWL) is a common metric for reporting weight loss after bariatric surgery. %EWL is one of the most accepted criteria for evaluating the success of bariatric surgery. It is calculated by the difference between the baseline weight and weight after surgery, divided by the difference between the baseline weight and the ideal weight, and expressed as a percentage: (initial weight − follow-up weight)/(initial weight − ideal weight for BMI 25) × 100 [22,23]. ^2^ IQR—interquartile range.

**Table 2:** Nutrient reference range values and deficiency/excess cut-off values.

Nutrient	Reference Range	Deficiency/Excess Cut-Off
Vitamin B12 (pg/mL)	187–883 pg/mL	<187 pg/mL
Folic acid (ng/mL)	2.2–17.5 ng/mL	<2.2 ng/mL
Vitamin D (ng/mL)	>30 ng/mL	<20 ng/mL
Calcium (mEq/L)	4.2–5.1 mEq/L	<4.2 mEq/L
PTH (pg/mL)	10–65 pg/mL	>65 pg/mL
Magnesium (mEq/L)	1.55–2.05 mEq/L	<1.55 mEq/L
Hemoglobin (g/dL)	12–16 g/dL	<12 g/dL
Iron (µg/dL)	49–151 µg/dL	<49 µg/dL
Ferritin (ng/mL)	Pre-menopause: 10–120 ng/mL Post-menopause: 10–250 ng/mL	<10 µg/dL
Transferrin (mg/dL)	200–360 mg/dL	>360 mg/dL

**Table 3:** Nutrient levels at the preoperative period and during the 12-month follow-up after RYGB and SG.

Nutrient/Type of Surgery	Before Surgery, Median (IQR)	*p*-Value	6 Months Post-Surgery, Median (IQR)	*p*-Value	12 Months Post-Surgery, Median (IQR)	*p*-Value
Vitamin B12 (pg/mL)		0.008		<0.001		<0.001
RYGB	348.00 (264.50–454.00)		270.50 (204.25–355.50)		271.00 (214.00–353.00)	
SG	407.00 (311.50–541.50)		372.50 (273.25–459.25)		382.00 (288.25–496.50)	
Folic acid (ng/mL)		0.434		0.357		0.022
RYGB	5.05 (3.90–7.20)		10.50 (7.00–15.10)		11.15 (6.30–15.28)	
SG	5.60 (4.00–7.90)		10.40 (5.95–14.35)		9.70 (5.00–14.10)	
Vitamin D (ng/mL)		0.597		0.329		0.696
RYGB	18.00 (12.00–24.00)		25.00 (19.00–31.00)		27.00 (21.00–33.00)	
SG	16.50 (13.00–21.00)		26.70 (21.00–30.00)		26.00 (20.00–33.75)	
Calcium (mEq/L)		0.218		0.002		0.001
RYGB	4.60 (4.50–4.80)		4.70 (4.60–4.90)		4.70 (4.60–4.90)	
SG	4.70 (4.60–4.80)		4.80 (4.70–4.90)		4.80 (4.70–4.90)	
PTH (pg/mL)		0.367		0.020		0.118
RYGB	49.80 (38.10–68.08)		45.20 (33.85–57.80)		44.10 (34.30–56.40)	
SG	51.30 (42.95–70.60)		40.70 (31.00–52.90)		40.05 (32.90–54.03)	
Magnesium (mEq/L)		0.849		0.064		0.863
RYGB	1.62 (1.54–1.69)		1.63 (1.54–1.71)		1.63 (1.55–1.70)	
SG	1.63 (1.52–1.69)		1.65 (1.57–1.74)		1.64 (1.56–1.71)	
Hemoglobin (g/dL)		0.001		0.010		0.005
RYGB	13.50 (12.90–14.20)		13.20 (12.60–13.90)		13.10 (12.43–13.98)	
SG	13.95 (12.90–15.00)		13.50 (12.80–14.40)		13.60 (12.70–14.23)	
Iron (µg/dL)		0.154		< 0.001		0.137
RYGB	74.00 (58.75–95.00)		78.00 (61.00–93.00)		91.00 (69.00–113.00)	
SG	88.00 (63.75–105.50)		91.50 (70.75–111.25)		96.00 (73.00–118.00)	
Ferritin (ng/mL)		0.011		<0.001		<0.001
RYGB	81.00 (39.30–141.70)		91.25 (48.48–179.60)		79.70 (33.38–153.68)	
SG	135.80 (60.80–276.00)		148.40 (71.95–258.95)		142.20 (67.90–243.70)	
Transferrin (mg/dL)		0.182		0.298		0.008
RYGB	274.00 (244.00–317.75)		235.50 (211.00–266.00)		251.00 (220.50–291.00)	
SG	269.00 (235.50–290.75)		230.00 (207.00–261.00)		237.00 (214.00–268.00)	

RYBG: Roux-en-Y gastric bypass; SG: sleeve gastrectomy; IQR: interquartile range.

**Table 4:** Prevalence of nutrient deficiencies/excess before surgery and in the 12-month follow-up period after surgery (RYGB vs. SG).

Nutrient/Type of Surgery	Before Surgery, *n* (%)	*p*-Value	6 Months Post-Surgery, *n* (%)	*p*-Value	12 Months Post-Surgery, *n* (%)	*p*-Value
Vitamin B12 (pg/mL)		0.684		<0.001		<0.001
RYGB	7 (5.60%)		44 (17.46%)		39 (16.74%)	
SG	2 (4.08%)		6 (4.69%)		1 (0.93%)	
Folic acid (ng/mL)		0.897		^a^		0.425
RYGB	3 (2.27%)		0 (0.00%)		2 (0.86%)	
SG	1 (1.96%)		0 (0.00%)		2 (1.87%)	
Vitamin D (ng/mL)		0.078		0.247		0.073
RYGB	103 (62.05%)		75 (29.64%)		52 (22.41%)	
SG	49 (74.24%)		31 (24.03%)		34 (31.48%)	
Calcium (mEq/L)		0.809		0.189		0.090
RYGB	10 (3.62%)		7 (2.79%)		6 (2.58%)	
SG	4 (3.15%)		1 (0.76%)		7 (6.31%)	
PTH (pg/mL)		0.546		0.039		0.528
RYGB	35 (26.12%)		41 (16.40%)		36 (15.86%)	
SG	15 (30.61%)		11 (8.66%)		14 (13.21%)	
Magnesium (mEq/L)		0.700		0.120		0.892
RYGB	85 (30.47%)		72 (28.35%)		58 (25.00%)	
SG	36 (28.57%)		28 (21.05%)		27 (24.32%)	
Hemoglobin (g/dL)		0.393		0.378		0.095
RYGB	25 (7.55%)		31 (11.79%)		39 (15.73%)	
SG	9 (5.49%)		12 (8.89%)		11 (9.32%)	
Iron (µg/dL)		0.620		0.119		0.318
RYGB	17 (12.69%)		28 (11.07%)		15 (6.41%)	
SG	6 (15.79%)		8 (6.15%)		4 (3.74%)	
Ferritin (ng/mL)		0.445		0.374		0.252
RYGB	2 (1.48%)		5 (1.97%)		10 (4.27%)	
SG	0 (0.00%)		1 (0.78%)		2 (1.83%)	
Transferrin (mg/dL)		0.075		0.149		0.017
RYGB	11 (9.17%)		4 (1.60%)		12 (5.15%)	
SG	0 (0.00%)		0 (0.00%)		0 (0.00%)	

RYBG: Roux-en-Y gastric bypass; SG: sleeve gastrectomy. ^a^ no statistics were computed because the values were constant.

## Data Availability

The original contributions presented in the study are included in the article material; further inquiries can be directed to the corresponding author.
